# Epidemiology of early infections and predictors of mortality after autologous hematopoietic stem-cell transplantation among multiple myeloma, Hodgkin, and non-Hodgkin lymphoma: the first experience from Palestine

**DOI:** 10.1186/s12879-022-07709-4

**Published:** 2022-09-07

**Authors:** Riad Amer, Husam Salameh, Sultan Mosleh, Adham Abu-Taha, Hamza Hamayel, Ahmad Enaya, Amro Adas, Ahmad Khursani, Mohamad Wild-Ali, Taghreed Mousa, Maher Battat, Aiman Daifallah, Amer Koni, Ramzi Shawahna

**Affiliations:** 1 Department of Medicine, An-Najah National University Hospital, P.O. Box 7, Nablus, Palestine; 2 Hematology and Oncology, An-Najah National University Hospital, Nablus, Palestine; 3 Department of Physiology, Pharmacology and Toxicology, Faculty of Medicine and Health Sciences, An-Najah National University, Nablus, Palestine; 4 Internal Medicine, An-Najah National University Hospital, Nablus, Palestine; 5 An-Najah BioSciences Unit, Centre for Poisons Control, Chemical and Biological Analyses, An-Najah National University, Nablus, Palestine

**Keywords:** Autologous hematopoietic stem cell transplantation, Hodgkin’s lymphoma, Non-Hodgkin’s lymphoma, Infection, Multiple myeloma, Mortality

## Abstract

**Background:**

Autologous hematopoietic stem-cell transplantation (HSCT) is the standard of care in many relapsed and refractory lymphoid malignancy, neuroblastoma, and multiple myeloma (MM). This study was conducted to describe the epidemiology of early infections that occurred within the first 100 days among patients who received HSCT for MM, Hodgkin (HL), and non-Hodgkin lymphoma (NHL) in Palestine.

**Methods:**

This study was conducted in a retrospective cohort design in the only autologous HSCT in Palestine in the period between 2014 and 2021. The medical records of the patients were reviewed to identify and collect demographic, clinical, and microbiological data on bacterial, viral, fungal, and parasitic infections as diagnosed by cultures, polymerase chain reaction, and fluorescent antibody testing.

**Results:**

A total of 145 patients were included in this study (median age = 44.0 [28.0, 53.5] years). Of those, 8 (5.5%) were younger than 18 years, 69 (47.6%) had MM, 53 (36.6%) had HL, and 23 (15.9%) had NHL. The source of fever had no focus in the majority of the cases 82 (56.6%), 12 (8.3%) had bloodstream infections, 8 (5.5%) had colitis, and 7.6 (5.0%) had pneumonia. Patients from whom gram-negative bacteria were isolated stayed in the hospital for longer duration compared to the other patients (median = 21.0 [19.0, 25.0] vs. 18.0 [15.0, 22.0] days, p-value = 0.043, respectively). The cumulative incidence of death in the first 100 days after infusion of stem cells was 3.4%. The cumulative incidence of death in the first 100 days post-transplantation was higher for patients with NHL compared to those with HL and MM (p-value = 0.017). Gram-negative and fungal infections were strong predictors of mortality.

**Conclusion:**

Bacterial gram-positive and gram-negative infections were the most common early infections among patients who underwent autologous HSCT for hematological malignancies (HM) in the only center in Palestine. The findings of this study are informative to healthcare providers and planners of care for patients who are scheduled to receive autologous HSCT for HM.

**Supplementary Information:**

The online version contains supplementary material available at 10.1186/s12879-022-07709-4.

## Introduction

Autologous hematopoietic stem-cell transplantation (HSCT) is the standard of care in many relapsed and refractory lymphoid malignancy, neuroblastoma, and multiple myeloma (MM) [1–3]. In Europe, more than half of all bone marrow transplants are autologous HSCT [3, 4]. Despite the use of various protocols in the treatment of myelomas, autologous HSCT remains one of the most commonly used therapeutic plans used to extend prognosis-free survival [3, 5]. In Arab countries, Hodgkin’s (HL) and non-Hodgkin’s lymphomas (NHL) were among the top 5 most prevalent cancers among the general population [6, 7].

Autologous HSCT was reported to be associated with some complications in the short and long term [8, 9]. One of the main complications of autologous HSCT is the incidence of infections [8–10]. Previous studies have shown that infections were associated with the doses of the chemotherapeutic agents used, degree of immunosuppression, length of neutropenia, damage to the mucous membranes in the gastrointestinal tract, and infections related to central or peripheral lines [11–13]. Infections occurring among patients who received autologous HSCT were shown to be associated with therapy-related mortality [14, 15].

Previous studies have shown that the majority of early infections that occur after autologous HSCT were caused by bacteria [16, 17]. Other infections were also caused by fungi, protozoa, and viruses. In Palestine, the epidemiology of infections and predictors of mortality after autologous HSCT among MM, HL, and NHL were not described before. Additionally, little is known about the prevalence rates of infections and transplantation-related mortality among patients who received autologous HSCT in Palestine. Moreover, microorganisms causing infections, treatments, and factors associated with infections and post-transplantation mortality were not well-recognized. It has been argued that identification of the microorganisms causing infections and the factors associated with higher risk for early infections and post-transplantation mortality might help design strategies to reduce the occurrence of these infections and reduce post-transplantation mortality rates. Therefore, providing insights into early infections among patients who receive autologous HSCT might inform locoregional implementation of infection control and prophylaxis policies in Palestine and other similar healthcare systems in developing and resource-limited countries.

This study was conducted to: (1) describe the epidemiology of early infections that occurred within the first 100 days among patients who received autologous HSCT for MM, HL, and NHL, (2) identify the microorganisms causing infections among the patients, (3) report the pharmacological agents used to treat these infections, (4) investigate associations between infections and different patient variables, and (5) identify predictors of post-transplantation mortality.

## Methods

### Study design

In this retrospective cohort study, all patients (*n* = *145*) with MM, HL, and NHL who underwent autologous HSCT in the period between June 2014 and February 2021 in the only center in Palestine were included.

### Patient preparation before autologous HSCT

To mobilize stem cells, patients were administered granulocyte colony-stimulating factor (G-CSF) (10 µg/kg/day) 4–5 days before stem cell collection [18]. The G-CSF was administered until the absolute neutrophil count (ANC) was above 1000 cells/µL for at least 2 consecutive days. Stem cells (CD34) were also chemo-mobilized using cyclophosphamide [19]. Peripheral blood samples were collected and blood apheresis was performed to obtain stem cells. The collected stem cells were cryopreserved at – 80 °C. Before the patients entered the bone marrow transplantation facility, complete blood count, liver function test, and kidney function test were obtained. Echocardiography and pulmonary function test were also obtained for each patient. Additionally, the patients were serologically screened for Epstein-Barr virus, Varicella-zoster virus, hepatitis B and C, and cytomegalovirus (CMV) viruses. Hepatitis B was screened for using hepatitis B surface antigen (HBsAg), hepatitis B antibodies (anti-HBs), total hepatitis B core antigen (anti-HBc), and immunoglobulin M (IgM). Hepatitis C was screened for using hepatitis C antibodies. The patients were screened for CMV using immunoglobulin G (IgG) and IgM. To prevent CMV infections, prophylactic use of antiviral drugs and preemptive therapy for the patients who develop signs of CMV infections during routine screening were used.

### The transplantation method

Patients with MM received melphalan conditioning chemotherapy [20]. Patients with lymphomas received lomustine, etoposide, ara-C (cytarabine), melphalan (LEAM), BCNU (carmustine), etoposide, ara-C (cytarabine), melphalan (BEAM), thiotepa, etoposide, ara-C (cytarabine), melphalan (TEAM), and/or melphalan conditioning chemotherapy [21]. Before infusion of the stem cells, patients received diazepam (1 mg), ondansetron (8 mg), and dexamethasone (8 mg) 1 h before infusion of stem cells to reduce the potential adverse effects. The cryopreserved stem cells were allowed to thaw in a sterile water bath (37–40 °C) bedside and were infused into the patients immediately through a central line.

### Prophylaxis

To minimize the risk of post-transplantation infections, the patients received prophylactic antimicrobial agents via the oral route. The antimicrobial agents were levofloxacin (750 mg) once daily, fluconazole (150 mg) once daily, and acyclovir (400 mg) twice daily. The patients received their prophylactic antimicrobial agents two days before the infusion of the stem cells until engraftment was achieved. In this study, white blood cells (WBCs) engraftment was defined as absolute neutrophilic count of more than 500 × 10^6^/L on 3 consecutive days and platelets engraftment was defined as platelet count of more than 20 × 10^9^/L on 3 consecutive days without a need for platelet transfusion [22, 23].

### Microbiological methods

The medical records of the patients were reviewed to identify and collect microbiological data on bacterial, viral, fungal, and parasitic infections as diagnosed by cultures, polymerase chain reaction, and fluorescent antibody testing [17]. An infection was defined as the isolation or detection of a microorganism with consistent signs and symptoms of the disease caused by that microorganism. Medical history, laboratory, and medical imaging reports were also used to define an infection. The bacteria isolated in this study were cultured on media. For *Staphylococcus epidermidis*, specimens were taken from two different sites using sterile technique. The two cultures were positive and the time for bacterial growth was less than 48 h. The invasive fungal infections including fungal pneumonia and fungal sinusitis were diagnosed based on computed tomography (CT) scan, serum galactomannan level, and clinical presentation. In this study, invasive fungal infections were generalized, systemic, deep-seated, severe, and life-threatening fungal infections, in contrast to more superficial, local, benign, self-limiting fungal infections [24]. The patients improved after administration of antifungal agents. Amoeba was diagnosed based on microscopic examination of stool. In this study, the first episode of infection was accounted for in the data analysis. Infections that occurred during the patient’s stay at the hospital after transplantation were included. We did not include any infections that could have occurred after the patient was discharged without complications or transferred to the primary/community care services.

### Mortality rate

The mortality rate was defined as the number of deaths in the population of the patients who received autologous HSCT scaled to the size of the population in the first 100 days [25].

### Statistical analysis

The data collected in this study were entered into IBM SPSS (v.21.0) for Windows (Armonk, New York). The data were expressed as numbers and percentages. Fisher’s Exact Test was used to compare groups. The cumulative incidence of death in the first 100 days post-transplantation was compared using Manel-Cox test and log-rank test for trend. Odds ratios with their corresponding 95% confidence intervals (95% CI) were computed in a multivariate logistic backward LR regression model in which variables with a p-value of < 0.05 in the Fisher's Exact Test were included. In this study, a p-value of < 0.05 indicated statistical significance.

### Ethical approval

This study was conducted in adherence to the international ethical principles and those in the Declaration of Helsinki. The study was approved by the Institutional Review Board of An-Najah National University. An-Najah National University Hospital approved this study. All patients provided written informed consents.

## Results

### Characteristics of the patients

A total of 145 patients underwent autologous HSCT in the period between June 2014 and February 2021. The median age of the patients who were included in this analysis was 44.0 [28.0, 53.5] years. Of those, 8 (5.5%) were younger than 18 years, 69 (47.6%) had MM, 53 (36.6%) had HL, and 23 (15.9%) had NHL. Of all patients, 81 (55.9%) were male, 32 (22.1%) had hypertension, 26 (17.9%) had diabetes mellitus, 11 (7.6%) had chronic heart disease, and 12 (8.3%) had chronic kidney disease. All patients who were younger than 18 years had HL. Hypertension was more prevalent among patients with MM (20.0%) compared to patients with NHL (1.4%) and HL (0.7%) (Pearson’s Chi-Square/Fisher’s exact test = 30.93, p-value < 0.001). Similarly, diabetes mellitus was more prevalent among patients with MM (15.9%) compared to patients with NHL (0.7%) and HL (1.4%) (Pearson’s Chi-Square/Fisher's exact test = 21.71, p-value < 0.001). Again, heart disease was more prevalent among patients with MM (6.9%) compared to patients with NHL (0.7%) (Pearson’s Chi-Square/Fisher’s exact test = 5.56, p-value = 0.017). Similarly, heart disease was more prevalent among patients with MM (7.6%) compared to patients with NHL (0.7%) (Pearson’s Chi-Square/Fisher’s exact test = 6.48, p-value = 0.011). None of the patients with HL had a heart or chronic kidney disease. Detailed demographic and clinical characteristics of the patients stratified by type of hematological malignancy (HM) are shown in Table 1.

**Table 1:** Demographic and clinical characteristics of the patients (*n* = *145*) stratified by type of HM

Variable	Type of HM						Chi-square/Fisher's exact test	p-value
	NHL		HL		MM			
	n	%	n	%	n	%		
Age (years)								
< 18	0	0.0	8	5.5	0	0.0	12.7	**0.001**
≥ 18	23	15.9	45	31.0	69	47.6		
Gender								
Male	14	9.7	25	17.2	42	29.0	2.6	0.273
Female	9	6.2	28	19.3	27	18.6		
Hypertension								
No	21	14.5	52	35.9	40	27.6	30.9	**< 0.001**
Yes	2	1.4	1	0.7	29	20.0		
Diabetes mellitus								
No	22	15.2	51	35.2	45	31.0	21.7	**< 0.001**
Yes	1	0.7	2	1.4	23	15.9		
Chronic heart disease								
No	22	15.2	53	36.6	59	40.7	5.6	**0.017**
Yes	1	0.7	0	0.0	10	6.9		
Chronic kidney disease								
No	22	15.2	53	36.6	58	40.0	6.5	**0.011**
Yes	1	0.7	0	0.0	11	7.6		

*HL* Hodgkin’s lymphoma, *HM* hematological malignancy, *NHL* non-Hodgkin’s lymphoma, *MM* multiple myeloma

Patients with HL were significantly younger than patients with NHL and MM. Patients with MM had significantly higher body weight compared to patients with HL and patients with NHL. Patients with HL were hospitalized for significantly shorter duration of time compared to patients with NHL and patients with MM. Details of the continuous variables of the patients stratified by their type of HM are shown in Table 2.

**Table 2:** Comparison between age, weight, and length of hospital stay of the patients (n = 145) stratified by their type of HM

Variable	Type of HM	Q1	Median (Q2)	Q3	p-value
Age (years)	NHL	33.0	43.0	51.0	**< 0.001**
	HL	18.0	24.0	32.0	
	MM	47.0	52.0	60.0	
Weight (kg)	NHL	54.5	69.0	89.0	**0.001**
	HL	55.0	65.0	76.0	
	MM	67.8	80.0	95.8	
Length of hospital stay	NHL	15.0	22.0	23.0	**0.023**
	HL	15.0	16.0	20.0	
	MM	16.0	19.0	22.0	

*HL* Hodgkin’s lymphoma, *HM* hematological malignancy, *NHL* non-Hodgkin’s lymphoma, *MM* multiple myeloma

### Details of the previous chemotherapy and radiotherapy

Patients with MM received bortezomib-based therapy, patients with HL received ABVD (doxorubicin, bleomycin, vinblastine, dacarbazine), and patients with NHL received CHOP (cyclophosphamide, hydroxydaunorubicin, oncovin (vincristine), prednisone/prednisolone). Additionally, patients with NHL also received ICE (ifosfamide, carboplatin and etoposide), IGEV (ifosfamide, gemcitabine, etoposide, vinorelbine), DHAP (dexamethasone, (H)igh-dose (A)ra-C-cytarabine, (P)latinol (cisplatin), and CVP (cyclophosphamide, vincristine, prednisone/prednisolone), respectively. Additionally, patients with HL also received ICE, IGEV, DHAP, and GDP (Gemcitabine, Dexamethasone, and Cisplatin). Concerning conditioning chemotherapy, all patients with MM received melphalan. Details of the previous chemotherapy and radiotherapy received by the patients who were included in this study are shown in Table 3.

**Table 3:** Details of the chemotherapy and radiotherapy received by the patients

Previous therapy	Type of HM						Chi-square/Fisher’s exact test	p-value
	NHL		HL		MM			
	n	%	n	%	n	%		
Radiotherapy								
No	20	13.8	47	32.4	62	42.8	0.2	0.720
Yes	3	2.1	6	4.1	7	4.8		
Bortezomib								
No	23	15.9	53	36.6	0	0.0	145.0	**< 0.001**
Yes	0	0.0	0	0.0	69	47.6		
ABVD								
No	23	15.9	0	0.0	69	47.6	145.0	**< 0.001**
Yes	0	0.0	53	36.6	0	0.0		
CHOP								
No	0	0.0	53	36.6	69	47.6	145.0	**< 0.001**
Yes	23	15.9	0	0.0	0	0.0		
ICE								
No	19	13.1	47	32.4	69	47.6	10.3	**0.002**
Yes	4	2.8	6	4.1	0	0.0		
IGEV								
No	14	9.7	26	17.9	69	47.6	44.7	**< 0.001**
Yes	9	6.2	27	18.6	0	0.0		
DHAP								
No	10	6.9	29	20.0	69	47.6	46.2	**< 0.001**
Yes	13	9.0	24	16.6	0	0.0		
CVP								
No	22	15.2	53	36.6	69	47.6	3.3	0.159
Yes	1	0.7	0	0.0	0	0.0		
GDP								
No	23	15.9	47	32.4	69	47.6	8.8	**0.005**
Yes	0	0.0	6	4.1	0	0.0		
Conditioning								
LEAM	16	11.0	37	25.5	0	0.0	100.6	**< 0.001**
BEAM	7	4.8	11	7.6	0	0.0		
TEAM	0	0.0	1	0.7	0	0.0		
Melphalan	0	0.0	4	2.8	69	47.6		

*HL* Hodgkin’s lymphoma, *HM* hematological malignancy, *NHL* non-Hodgkin’s lymphoma, *MM* multiple myeloma

### Source of fever

In this study, the source of fever had no focus in the majority of the cases (56.6%). Those patients likely had febrile neutropenia. The source of fever in 5 patients (3.4%) had more than one focus. Detailed sources of fever in the patients included in this study are shown in Table 4.

**Table 4:** Detailed sources of fever in the patients included in this study

Source of fever	N	%	Duration of fever (days)			
			Q1	Median (Q2)	Q3	p-value
No focus	82	56.6	2.5	4.0	7.0	**0.012**
Bloodstream	12	8.3	3.3	8.5	12.8	
Colitis	8	5.5	5.3	8.5	11.8	
Pneumonia	11	7.6	5.0	10.0	13.0	
Urinary tract infection	2	1.4	–	–	–	–
Ear infection	2	1.4	–	–	–	–
Tonsillitis	1	0.7	–	–	–	–
Typhlitis	1	0.7	–	–	–	–
Skin	1	0.7	–	–	–	–
Bloodstream and colitis	1	0.7	–	–	–	–
Bloodstream, pneumonia, and urinary tract infection	1	0.7	–	–	–	–
Urinary tract infection and pneumonia	1	0.7	–	–	–	–
Bloodstream and pneumonia	1	0.7	–	–	–	–
Colitis and sinusitis	1	0.7	–	–	–	–

The duration of fever in patients who had blood stream infections, colitis, and pneumonia was significantly longer than those who had fever of no focus as shown in Table 4.

### The microorganisms isolated or identified

In this study, a total of 43 microorganisms were isolated or identified in 34 (23.4%) patients. Microorganisms were isolated or identified from 15/66 patients with MM, 12/53 patients with HL, and 7/23 patients with NHL. Of the isolated or identified microorganisms, 10.3% were gram-negative bacteria, 13.1% were gram-positive bacteria, 2.8% were fungi, and 1.4% were either protozoa or viruses. Details of the microorganisms isolated or identified in this study are shown in Table 5.

**Table 5:** Details of the microorganisms isolated or identified in this study

Microorganism	n	% of all infections	% of patients
Gram-positive bacteria			
* Clostridium difficile*	9	20.9	26.5
* Staphylococcus epidermidis*	2	4.7	5.9
* Enterococcus faecalis*	2	4.7	5.9
* Streptococcus mitis*	1	2.3	2.9
* Staphylococcus capitis*	1	2.3	2.9
* Streptococcus oralis*	1	2.3	2.9
Gram-negative bacteria			
* Escherichia coli*	9	20.9	26.5
* Klebsiella pneumoniae*	6	14.0	17.6
* Pseudomonas aeruginosa*	3	7.0	8.8
* Haemophilus influenzae*	1	2.3	2.9
* Acinetobacter lwoffii*	1	2.3	2.9
* Acinetobacter baumannii*	1	2.3	2.9
Fungi			
Fungal pneumonia	2	4.7	5.9
Fungal sinusitis	1	2.3	2.9
Candida CLABSI	1	2.3	2.9
Protozoa			
Amoeba	1	2.3	2.9
Virus			
Coronavirus disease of 2019	1	2.3	2.9

*CLABSI* central line-associated bloodstream infection

There was one case of multidrug resistant *Acinetobacter baumannii*, the other microorganisms responded to treatment. Significantly less gram-positive bacteria were isolated from patients who received DHAP (Chi-Square/Fisher’s exact test = 7.4, p-value = 0.004). The median number of CD34 cells collected from the patients from whom gram-positive bacteria were isolated was significantly less than that those collected from the other patients (median = 11.0 [10.0, 14.0] × 10^6^ cells vs. 12.0 [11.0, 15.0] × 10^6^ cells, p-value = 0.015). Similarly, WBCs engraftment was significantly longer in patients from whom gram-positive bacteria were isolated compared to the other patients (median = 13.0 [12.0, 15.0] days vs. median = 12.0 [10.5, 14.] days, p-value = 0.034). Patients from whom gram-negative bacteria were isolated had significantly less WBCs and ANC at discharge compared to the other patients (median = 3.3 [2.5, 5.6] × 10^3^ cells/µL vs. 5.4 [3.5, 7.3] × 10^3^ cells/µL, p-value = 0.017 and median = 1.9 [1.4, 3.7] × 10^3^ cells/µL vs. 3.7 [2.1, 5.3] × 10^3^ cells/µL, p-value = 0.032, respectively). Patients from whom gram-negative bacteria were isolated stayed in the hospital for longer duration compared to the other patients (median = 21.0 [19.0, 25.0] days vs. 18.0 [15.0, 22.0] days, p-value = 0.043, respectively). In this study, there was a significant association between receiving ceftazidime and having *Clostridium difficile* (Chi-Square/Fisher’s exact test = 6.9, p-value = 0.035). Details of the treatments administered to patients are shown in Additional file 1: Table S1.

Patients with NHL had significantly more fungal pneumonia infections compared to patients with HL and MM (Chi-Square/Fisher’s exact test = 6.5, p-value = 0.024). Similarly, patients who received CHOP had significantly more fungal pneumonia infections compared to patients who did not receive CHOP (Chi-Square/Fisher’s exact test = 10.7, p-value = 0.024). On the other hand, patients who received IGEV had significantly more *Klebsiella pneumoniae* infections compared to the patients who did not receive IGEV (Chi-Square/Fisher’s exact test = 5.9, p-value = 0.034). Other variables of the patients were not significantly associated with early infections. Prevalence of early infections was not significantly associated with other variables of the patients.

### Mortality rate

**Fig. 1:**
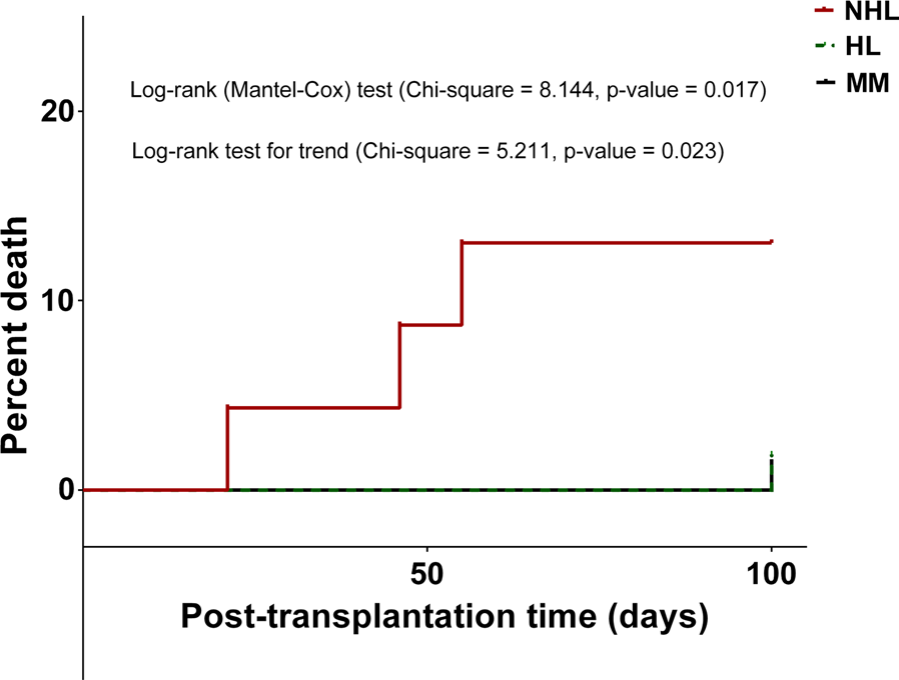
Cumulative incidence of death in the first 100 days post-transplantation. *HL* Hodgkin’s lymphoma, *NHL* non-Hodgkin’s lymphoma, *MM* multiple myeloma

The overall mortality rate in the first 100 days after infusion of stem cells was 3.4%. The death rates were 3/23 with NHL, 1/53 with HL, and 1/66 with MM. The 5 patients who died in this study had infections and died as a result of these infections. The cumulative incidence of death in the first 100 days post-transplantation in the three groups is shown in Fig. 1. The cumulative incidence of death for patients with NHL differed significantly (p-value < 0.05) from those with HL and MM as indicated by the Mantel-Cox test and log-rank test for trend.

### Prevalence and factors associated with mortality

The post-transplantation mortality was significantly associated with being infected with *Escherichia coli*, *Klebsiella pneumoniae*, gram-negative bacteria, receiving amikacin, voriconazole, colistin, and caspofungin. Details of these associations are shown in Table 6.

**Table 6:** Associations between post-transplantation mortality with infections and treatments

Infection/treatment	N	%	Post-transplantation mortality				Fisher’s exact test	p-value
			No		Yes			
			n	%	n	%		
Microorganism								
* Escherichia coli*								
No	136	93.8	133	91.7	3	2.1	10.1	**0.031**
Yes	9	6.2	7	4.8	2	1.4		
* Klebsiella pneumoniae*								
No	139	95.9	136	93.8	3	2.1	16.7	**0.014**
Yes	6	4.1	4	2.8	2	1.4		
* Staphylococcus epidermidis*								
No	143	98.6	139	95.9	4	2.8	13.1	0.068
Yes	2	1.4	1	0.7	1	0.7		
Fungal pneumonia								
No	143	98.6	139	95.9	4	2.8	13.1	0.068
Yes	2	1.4	1	0.7	1	0.7		
Gram-negative bacteria								
No	130	89.7	128	88.3	2	1.4	13.7	**0.008**
Yes	15	10.3	12	8.3	3	2.1		
Treatment								
Amikacin								
No	47	32.4	43	29.7	4	2.8	5.3	**0.038**
Yes	98	67.6	97	66.9	1	0.7		
Voriconazole								
No	126	86.9	124	85.5	2	1.4	9.9	**0.016**
Yes	19	13.1	16	11.0	3	2.1		
Colistin								
No	114	78.6	113	77.9	1	0.7	10.5	**0.008**
Yes	31	21.4	27	18.6	4	2.8		
Caspofungin								
No	133	91.7	131	90.3	2	1.4	18.1	**0.004**
Yes	12	8.3	9	6.2	3	2.1		

Multivariate logistic regression showed that patients who had gram-negative infections were 16-fold (95% CI: 2.4–105.3, β = 2.8, p-value = 0.004) more likely to experience mortality compared to patients who did not have gram-negative infections. On the other hand, patients who had fungal infections and received voriconazole and caspofungin were 19.9-fold (95% CI: 1.8–222.1, β = 3.0, p-value = 0.015) and 36.2-fold (95% CI: 3.2–406.9, β = 3.6, p-value = 0.004) more likely to experience mortality compared to patients who did not have fungal infections and did not receive these treatments. Details of the multivariate logistic regression model are shown in Additional file 1: Table S2.

## Discussion

Early infections are common after autologous HSCT in different healthcare systems. In this study, the epidemiology of early infections among patients who received autologous HSCT for MM, HL, and NHL was described for the first time in the only center in Palestine. Additionally, predictors of mortality were identified. The findings of this study would help establish infections data that might be used in screening and auditing post-transplantations in the Palestinian healthcare system and other similar healthcare systems around the world. Additionally, the findings of this study are informative to healthcare providers, decision-makers and other stakeholders who might be interested in developing measures that would reliably predict patients who are at higher risk for infection and death after autologous HSCT.

In this study, the majority of early infections among patients who underwent autologous HSCT were bacterial gram-positive and gram-negative infections. *Clostridium difficile* and *Escherichia coli* were the most prevalent gram-positive and gram-negative microorganisms, respectively. The findings reported in this study were consistent with those reported in regional studies in Saudi Arabia and Lebanon and those reported in St. Jude Children’s Research Hospital [17, 26, 27]. Additionally, *Clostridium difficile* infections were shown to be prevalent among patients who underwent autologous HSCT in Johns Hopkins Hospital in the US and Hôpital Maisonneuve-Rosemont in Canada [11]. In this study, the patients who had gram-negative infections had low CD34, WBCs, and ANC, and long WBCs engraftment. These findings indicate that their immune system was compromised, hence, more susceptible to infections. Therefore, it was not surprising that patients who had gram-negative infections had long hospital stays. These findings indicate that healthcare providers should take into account the modifiable risk factors that can be associated with infections.

In this study, 2.8% of the patients had early fungal infections. Previous studies have shown that the risk of fungal infections among patients who underwent autologous HSCT was low [10, 28]. In this study, receiving CHOP was associated with fungal pneumonia infections, and receiving IGEV was associated with Klebsiella pneumoniae infections. These findings could be explained by the immunosuppressant effect of these chemotherapeutic agents. Therefore, healthcare providers should consider using higher doses of prophylactic antifungal therapy for the patients scheduled to receive these chemotherapeutic protocols [10]. Other than COVID-19, no viral infections were identified among the patients who underwent autologous HSCT in this study. The findings were consistent with those reported in a recent study in Saudi Arabia [17]. These findings could be explained by the recent improvements in screening measures to ensure serological clearance before transplantation and the use of prophylactic antiviral agents. The patients included in this study were screened for human immunodeficiency virus, varicella zoster, hepatitis, syphilis, brucella, toxoplasmosis, cytomegalovirus, Epstein-Barr virus, herpes simplex virus, and human T-cell lymphotropic virus. Taken together, these findings might indicate that screening to ensure serological clearance and the use of prophylactic antiviral agents are effective in preventing early viral infections among patients who received autologous HSCT.

In this study, the 5 patients who died had infections. This mortality rate could be the infection-associated post-transplantation mortality rate. The cumulative incidence of death in the first 100 days post-transplantation was low. Curve analysis showed a high mortality rate among patients with NHL compared to those with HL and MM. Previous studies showed variable survival rates among patients with HM after undergoing autologous HSCT [29–31]. In this study, the multivariate analysis showed that gram-negative infection was a strong predictor of the first 100 days post-transplantation mortality. Additionally, fungal infections and receiving antifungal agents were also predictors of post-transplantation mortality. The findings reported in this study indicate that healthcare providers should consider measures to reduce the incidence of early gram-negative and fungal infections among patients who undergo autologous HSCT.

### Strengths and limitations of the study

This study has several strengths and limitations that should be considered while interpreting the results. First, this is the first study on the epidemiology of early infections and predictors of mortality after autologous HSCT among patients with HM in the Palestinian healthcare system. Establishing epidemiological data on early infections among this vulnerable subset of immunocompromised patients. These data can be informative to healthcare providers, decisions makers, and other stakeholders who are interested in designing measures to reduce early infections among patients with HM who undergo autologous HSCT. Second, correlations were established between infections, length of hospital stay, and mortality. It has been argued that developing reliable predictive measures for infections and patient outcomes is highly useful in triaging patients who could be at higher or lower risk for early infections, longer hospital stays, and mortality. Third, the patients included in this study were diverse in terms of gender, age group, co-morbidities, type of HM, and chemotherapy protocol. This diversity should have improved the representativeness of the sample included in this study. The data derived from representative samples are more reliable and have higher external validity.

On the other hand, the study had some limitations. First, the study was conducted in a retrospective design. Compared to those generated from retrospective studies, data generated from prospective studies are more reliable. Second, the number of patients included in this study was relatively small. It is noteworthy mentioning that An-Najah National University Hospital was established in 2013 and autologous HSCT services were recently introduced to the Palestinian healthcare system. Traditionally, patients with HM were referred to other countries to receive autologous HSCT. With the increasing number of HM that need autologous HSCT services future prospective studies could be feasible. Third, many patients who could have developed infections after autologous HSCT might have received treatment elsewhere. Data relevant to those patients were not collected in this study. Therefore, infections reported in this study might have been underestimated.

## Conclusion

In this first retrospective study, bacterial gram-positive and gram-negative infections were the most common early infections among patients who underwent autologous HSCT for HM in the only center in Palestine. Patients with gram-negative bacteria had low CD34, WBCs, ANC, and long WBCs engraftment. Gram-negative bacteria and fungal infections were strong predictors of post-transplantation mortality. The findings of this study are informative to healthcare providers and planners of care for patients who are scheduled to receive autologous HSCT for HM.

## Supplementary Information

**Additional file 1.** **Table S1.** Detailed treatments used in this study. **Table S2.** Details of the multivariate logistic regression model.

## Data Availability

The datasets used and/or analyzed during the current study are available from the corresponding authors on reasonable request.
