# Supplementary material for: Epidemiology of early infections and predictors of mortality after autologous hematopoietic stem-cell transplantation among multiple myeloma, Hodgkin, and non-Hodgkin lymphoma: the first experience from Palestine
Source: BMC Infect Dis. 2022 Sep 7;22:725. doi: 10.1186/s12879-022-07709-4 (PMC9449926; doi:10.1186/s12879-022-07709-4)
Supplement: Supplementary file 1 — Additional file 1. Table S1. Detailed treatments used in this study. Table S2. Details of the multivariate logistic regression model. [file 12879_2022_7709_MOESM1_ESM.docx]

**Supplementary materials for:**

**Epidemiology of early infections and predictors of mortality after** **autologous hematopoietic stem-cell transplantation among multiple myeloma, Hodgkin, and non-Hodgkin lymphoma: the first experience from Palestine**

Riad Amer^1,2*^, Husam Salameh^1,2*^, Sultan Mosleh^1,2^, Adham Abu-Taha^3^, Hamza Hamayel^4^, Ahmad Enaya^4^, Amro Adas^4^, Ahmad Khursani^2^, Mohamad Wild-Ali^2^, Taghreed Mousa^2^, Maher Battat^2^, Aiman Daifallah^5^, Amer Koni^5^, Ramzi Shawahna^3,6^

^1^Department of Medicine, An-Najah National University Hospital, Nablus, Palestine

^2^Hematology and Oncology, An-Najah National University Hospital, Nablus, Palestine

^3^Department of Physiology, Pharmacology and Toxicology, Faculty of Medicine and Health Sciences, An-Najah National University, Nablus, Palestine

^4^Internal Medicine, An-Najah National University Hospital, Nablus, Palestine

^5^Internal Medicine, An-Najah National University Hospital, Nablus, Palestine

^6^An-Najah BioSciences Unit, Centre for Poisons Control, Chemical and Biological Analyses, An-Najah National University, Nablus, Palestine

**^*^Correspondence:**

Dr. Riad Amer (riad.amer@outlook.com), Dr. Hussam Salameh ([husam.salameh@hotmail.com](mailto:husam.salameh@hotmail.com)), Department of Medicine, An-Najah National University Hospital, Nablus, Palestine, An-Najah National University Hospital, Nablus, P.O. Box 7, Nablus, Palestine

## Treatments administered to patients who received autologous hematopoietic stem cell transplantation

More than half of the patients who received autologous HSCT in this study received piperacillin/tazobactam, amikacin, carbapenems, and vancomycin as both empiric and definitive treatment. Details of the treatments administered to the patients in this study are provided in Supplementary Table S1.

**Table S1:** Detailed treatments used in this study

| **Antibiotic** | **n** | **% of patients** |
| --- | --- | --- |
| Piperacillin/tazobactam | 102 | 70.3 |
| Amikacin | 98 | 67.6 |
| Carbapenems | 88 | 60.7 |
| Vancomycin | 84 | 57.9 |
| Colistin | 31 | 21.4 |
| Voriconazole | 19 | 13.1 |
| Caspofungin | 13 | 9.0 |
| Ceftazidime | 13 | 9.0 |
| Teicoplanin | 12 | 8.3 |
| Metronidazole | 7 | 4.8 |
| Tigecycline | 2 | 1.4 |
| Gentamicin | 1 | 0.7 |

Patients who had *Escherichia coli* and *Klebsiella pneumoniae* received carbapenems. Similarly, patients who had *Escherichia coli* and other gram-negative bacteria received colistin. Patients who had fungal pneumonia received voriconazole and patients who had fungal sinusitis received caspofungin and voriconazole.

**Table S2:** Details of the multivariate logistic regression model

|  |  |  |  |  |  |  | **95% CI for OR** | |
| --- | --- | --- | --- | --- | --- | --- | --- | --- |
| **Step** | **Variable** | **β** | **SE** | **Wald** | **p-value** | **OR** | **Lower** | **Upper** |
|  | **Microorganism** |  |  |  |  |  |  |  |
| 1 | *Escherichia coli* | 0.1 | 1.6 | 0.0 | 0.945 | 1.1 | 0.05 | 25.95 |
|  | *Klebsiella pneumoniae* | 1.5 | 1.5 | 1.0 | 0.317 | 4.3 | 0.25 | 74.15 |
|  | *Staphylococcus epidermidis* | 1.7 | 3.3 | 0.3 | 0.615 | 5.2 | 0.01 | 3235.92 |
|  | Fungal pneumonia | 1.7 | 3.3 | 0.3 | 0.615 | 5.2 | 0.01 | 3235.92 |
|  | Gram-negative bacteria | 2.0 | 1.5 | 1.7 | 0.193 | 7.4 | 0.36 | 153.07 |
|  | Constant | -4.3 | 0.7 | 34.6 | **< 0.001** | 0.0 |  |  |
| 2 | *Klebsiella pneumoniae* | 1.4 | 1.4 | 1.0 | 0.310 | 4.2 | 0.26 | 66.06 |
|  | *Staphylococcus epidermidis* | 1.7 | 3.2 | 0.3 | 0.605 | 5.3 | 0.01 | 3022.14 |
|  | Fungal pneumonia | 1.7 | 3.2 | 0.3 | 0.605 | 5.3 | 0.01 | 3022.14 |
|  | Gram-negative bacteria | 2.1 | 1.2 | 3.2 | 0.074 | 8.0 | 0.82 | 78.08 |
|  | Constant | -4.3 | 0.7 | 34.6 | **< 0.001** | 0.0 |  |  |
| 3 | *Klebsiella pneumoniae* | 1.5 | 1.4 | 1.2 | 0.268 | 4.6 | 0.31 | 66.78 |
|  | Fungal pneumonia | 2.5 | 2.1 | 1.3 | 0.247 | 11.8 | 0.18 | 772.63 |
|  | Gram-negative bacteria | 2.1 | 1.2 | 3.3 | 0.070 | 8.2 | 0.84 | 79.05 |
|  | Constant | -4.3 | 0.7 | 34.3 | **< 0.001** | 0.0 |  |  |
| 4 | Fungal pneumonia | 3.0 | 1.8 | 2.7 | 0.099 | 19.5 | 0.57 | 663.95 |
|  | Gram-negative bacteria | 2.6 | 1.0 | 6.7 | **0.010** | 13.4 | 1.87 | 96.02 |
|  | Constant | -4.3 | 0.7 | 33.5 | **< 0.001** | 0.0 |  |  |
| 5 | Gram-negative bacteria | 2.8 | 1.0 | 8.3 | **0.004** | 16.0 | 2.43 | 105.33 |
|  | Constant | -4.2 | 0.7 | 34.1 | **< 0.001** | 0.0 |  |  |
|  | **Treatment** |  |  |  |  |  |  |  |
| 1 | Amikacin | -1.8 | 1.4 | 1.8 | 0.183 | 0.2 | 0.01 | 2.35 |
|  | Voriconazole | 2.8 | 1.4 | 3.9 | **0.047** | 16.7 | 1.04 | 269.46 |
|  | Colistin | 0.8 | 1.4 | 0.3 | 0.583 | 2.2 | 0.14 | 34.07 |
|  | Caspofungin | 3.1 | 1.6 | 4.1 | **0.044** | 23.1 | 1.09 | 489.74 |
|  | Constant | -4.9 | 1.4 | 12.8 | **< 0.001** | 0.0 |  |  |
| 2 | Amikacin | -1.9 | 1.4 | 2.0 | 0.155 | 0.1 | 0.01 | 2.07 |
|  | Voriconazole | 3.1 | 1.3 | 5.2 | **0.023** | 21.5 | 1.54 | 301.63 |
|  | Caspofungin | 3.6 | 1.3 | 7.2 | **0.007** | 36.8 | 2.63 | 513.51 |
|  | Constant | -4.7 | 1.3 | 13.7 | **< 0.001** | 0.0 |  |  |
| 3 | Voriconazole | 3.0 | 1.2 | 5.9 | **0.015** | 19.9 | 1.78 | 222.14 |
|  | Caspofungin | 3.6 | 1.2 | 8.4 | **0.004** | 36.2 | 3.21 | 406.85 |
|  | Constant | -5.4 | 1.1 | 22.1 | **< 0.001** | 0.0 |  |  |

CI: confidence interval, OR: odds ratio, SE: standard error
